# Bed rest decreases resting skeletal muscle O_2_ uptake and resting energy expenditure in young and elderly subjects

**DOI:** 10.1113/EP092709

**Published:** 2026-04-02

**Authors:** Giovanni Baldassarre, Simone Porcelli, Lucrezia Zuccarelli, Lorenza Brocca, Noemi Arboit, Laura Gobbo, Michele Lacerenza, Alessio Marciano, Chiara Motto, Giulia Sanguin, Mladen Gasparini, Boštjan Šimunič, Rado Pišot, Marco Narici, Bruno Grassi

**Affiliations:** ^1^ Department of Medicine University of Udine Udine Italy; ^2^ Department of Molecular Medicine University of Pavia Pavia Italy; ^3^ PIONIRS S.r.l. Milano Italy; ^4^ Splošna Bolnišnica Izola Izola Slovenia; ^5^ Science and Research Centre Institute of Kinesiology Research Koper Slovenia; ^6^ Department of Biomedical Sciences University of Padova Padova Italy

**Keywords:** disuse, inactivity, oxidative metabolism, resting metabolic rate, skeletal muscle

## Abstract

A decrease in resting muscle O_2_ uptake (${\dot{V}}_{O_{2}m}$) described during bed rest (BR) could determine a decreased whole‐body resting energy expenditure (REE), potentially useful during prolonged spaceflights. Two groups of recreationally active men (young [Y], *n* = 8, age 23 ± 5 years; elderly [E], *n* = 10, age 69 ± 3 years) underwent a 21‐day (Y) or a 10‐day (E) horizontal BR without countermeasures. Measurements were performed before and post‐BR. Fat‐free mass (FFM) was measured by bioimpedance analysis; REE was measured by open‐circuit spirometry; resting ${\dot{V}}_{O_{2}m}$ was measured by time‐domain near‐infrared spectroscopy in the vastus medialis during a transient ischaemia; citrate synthase (CS) activity (estimate of mitochondrial volume) was measured on a vastus lateralis muscle biopsy sample. FFM decreased significantly both in Y (−8%, *P *< 0.001) and in E (−5%, *P* = 0.009). Both resting ${\dot{V}}_{O_{2}m}$ (−45%, −2.1% day^−1^ in Y, *P* = 0.025; −29%, −2.9% day^−1^ in E, *P* = 0.001) and REE (−15%, −0.7% day^−1^ in Y, *P* = 0.012; −12%, −1.2% day^−1^ in E, *P* = 0.012) decreased during BR. CS activity decreased in E (−31%, *P* = 0.005), whereas the decrease in Y (−12%) did not reach statistical significance (*P* = 0.38). After resting, ${\dot{V}}_{O_{2}m}$ data normalized for CS activity values in post‐ versus pre‐BR were lower in Y (*P* = 0.021) but not in E (*P* = 0.99). The decreased resting ${\dot{V}}_{O_{2}m}$ and REE may represent a ‘recalibration’ of ATP supply to a reduced ATP demand, aimed at preventing excessive reactive oxygen species production and muscle atrophy. It would mitigate biological and logistic challenges of prolonged spaceflights, but could negatively impact the health status of the subjects.

## Abstract (highlights)

- **What is the central question of this study?**
  Is bed rest (BR), in young (Y) and elderly (E) subjects, associated with a decreased resting muscle O_2_ uptake (${\dot{V}}_{O_{2}m}$) and with a decreased whole‐body resting energy expenditure (REE)?
- **What is the main finding and its importance?**
  Both resting ${\dot{V}}_{O_{2}m}$ and REE decreased during BR, both in Y (21‐day BR) and in E (10‐day BR). These inactivity‐related decreases, possibly aimed at preventing excessive reactive oxygen species production and muscle atrophy, would mitigate biological and logistic challenges in prolonged spaceflights, but could also alter body mass homeostasis and increase the risk of metabolic diseases.

## 1 INTRODUCTION

In individuals with low levels of physical activity the resting energy expenditure (REE) constitutes approximately 60% of the total daily energy expenditure, making it a primary contributor to overall energy usage (Fernandez‐Versejo et al., 2024; Heymsfield et al., 2021). Among the key factors influencing REE, skeletal muscle mass plays a pivotal role (Fernandez‐Verdejo et al., 2024; Heymsfield et al., 2021). Both these variables (skeletal muscle mass and REE) decline with age (Heymsfield et al., 2021). While an elevated REE is generally considered beneficial in terms of the prevention of obesity and the maintenance of body mass (BM) (Heymsfield et al., 2021), there are specific circumstances in which a reduced REE could provide distinct advantages. A reduced REE could be beneficial during long‐duration spaceflights, since it could reduce the rates of crewmember consumable use (food, water and O_2_) and CO_2_ production (Regan et al., 2020). This concept led these authors to envisage the induction (by sleep interventions, pharmacological sedation, etc.) of a state of shallow metabolic depression during spaceflights (Regan et al., 2020). Even by being far less pronounced compared to the typical reduction of REE (>90%) observed in obligate hibernating animals (Staples et al., 2022), a condition of shallow metabolic depression could reduce REE by ∼20%, allowing a mitigation of numerous biological and logistic challenges of prolonged spaceflights (Regan et al., 2020).

A decrease in REE could spontaneously happen in humans exposed to the profound inactivity and simulated microgravity associated with bed rest (BR). BR studies are frequently employed to simulate, on Earth, some of the physiological adjustments to microgravity encountered during spaceflights (Ade et al., 2017; Pavy‐Le Traon et al., 2007; Reid‐Larsen et al., 2017). In a recent BR campaign, our group has described a ∼25% decrease of resting O_2_ uptake by skeletal muscles (resting ${\dot{V}}_{O_{2}m}$) in a different cohort of young subjects exposed to a 10‐day horizontal BR (Zuccarelli et al., 2021). If we consider that skeletal muscles represent ∼40% of total body mass, a reduced resting ${\dot{V}}_{O_{2}m}$ should translate into a decreased whole‐body REE. Decreased resting ${\dot{V}}_{O_{2}m}$ and REE following bed rest could be a sign of a ‘recalibration’ of ATP supply to reduced ATP demand, possibly aimed at preventing excessive reactive oxygen species (ROS) production and attenuating muscle atrophy (Delfinis et al., 2025).

Decreased resting ${\dot{V}}_{O_{2}m}$ and REE would be of interest also in terms of the adaptations to periods of profound inactivity and muscle disuse, frequently encountered on Earth in cases of hospitalizations, intercurrent diseases, environmental constraints, etc. BR studies indeed allow the study of both microgravity and profound inactivity/muscle disuse. The utilization of a horizontal BR approach (and not the −6° head‐down tilt approach; see Pavy‐Le Traon et al., 2007), as done in the present study (as well as in several previous studies by our group; see e.g. Baldassarre et al., 2022; Zuccarelli et al., 2021, 2025), shifts the interest of the study slightly more towards inactivity/muscle disuse.

The main aim of the present study was to verify and quantify the decrease in REE in young subjects exposed to BR, in association with the reduced resting ${\dot{V}}_{O_{2}m}$. Secondary aims were to answer the following questions: after the initial 10 days, do REE and resting ${\dot{V}}_{O_{2}m}$ continue to decrease during BR periods of longer duration? What happens to REE and resting ${\dot{V}}_{O_{2}m}$ in elderly subjects exposed to BR? Are the REE and resting ${\dot{V}}_{O_{2}m}$ decreases associated with the decreases in muscle mass and mitochondrial volume frequently observed during BR? We therefore hypothesized a decreased REE associated with a decreased resting ${\dot{V}}_{O_{2}m}$ following a relatively short BR period, occurring both in young and in elderly subjects.

## 2 METHODS

### 2.1 Subjects

Two groups of recreationally active men participated in this study: one group was composed of young (Y) subjects (*n* = 8, age 23 ± 5 [mean ± SD] years), and the other group was composed of elderly (E) subjects (*n* = 10; age 69 ± 3 years). Subjects were informed about the aims, procedures and possible risks of the investigations before giving their written informed consent to participate. The study was approved by the National Committee for Medical Ethics at the Ministry of Health of the Republic of Slovenia (Ref. no. 0120‐123/2023/9, 21 July 2023) and conformed to the *Declaration of Helsinki* (2000).

None of the participants was engaged in competitive sports activities or followed specific training programmes before the study. Subjects underwent a medical screening before the study. Exclusion criteria were: regular smoking; habitual use of drugs; blood clotting defects or history of deep vein thrombosis with D‐dimer values *>*500 µg L^−1^; acute or chronic skeletal, neuromuscular, metabolic and cardiovascular disease conditions; previous history of embolism, inflammatory diseases, psychiatric disorders, epilepsy or presence of ferromagnetic implants.

During the BR period subjects consumed an individually controlled, standardized eucaloric diet and were allowed to drink water or unsweetened tea ad libitum. The dietary energy requirement was designed for each subject by multiplying resting energy expenditure (calculated by using the FAO/WHO equation and fat‐free mass and fat mass data obtained by bioelectrical impedance; Müller et al., 2004) by a factor 1.2 (Biolo et al., 2008). The macronutrient food content was set at 60% carbohydrates, 25% fats and 15% proteins.

**FIGURE 1:**
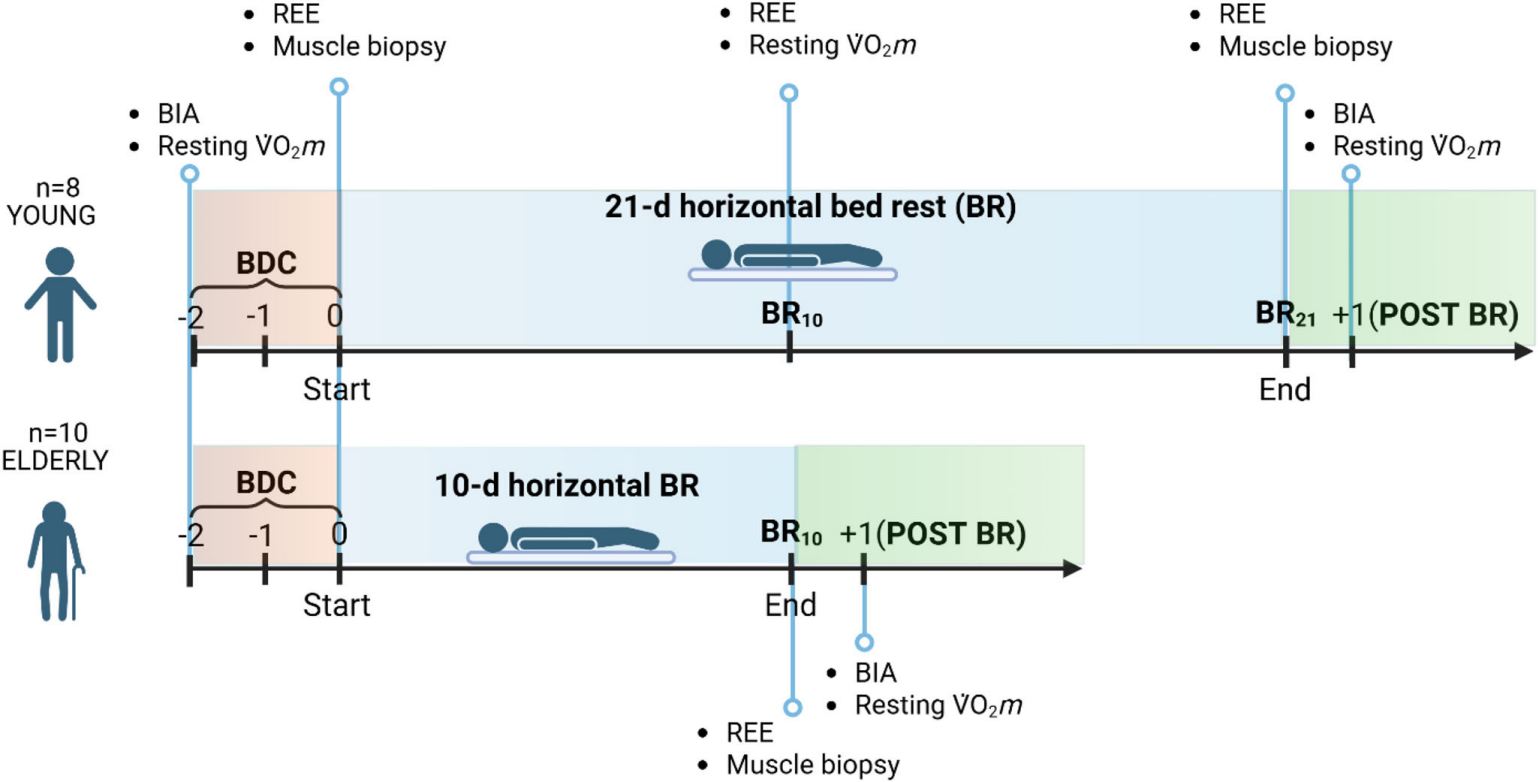
Schematic representation of the bed rest (BR) exposure and of the timing of performed measurements for the two groups of subjects, young (Y) and elderly (E). BDC, baseline data collection; BIA, bioimpedance analysis; REE, resting energy expenditure. Created with BioRender.com.

Subjects were tested before or at the beginning of BR (baseline data collection, BDC) and at the end or immediately after a 21‐day (Y) or 10‐day (E) horizontal BR without countermeasures, carried out at the General Hospital of Izola, Slovenia. In Y some measurements were also carried out after 10 days of BR. A schematic representation of the BR exposure and of the timing of performed measurements is shown for Y and E in Figure 1. During BR neither deviations from the lying position nor muscle stretching or static contractions were allowed. Adherence to the assigned protocol was ensured by continuous closed‐circuit video surveillance and constant supervision by researchers and medical staff.

### 2.2 Measurements

The environmental conditions within the hospital rooms in which measurements were performed remained stable throughout the experimental sessions. Before the data collection subjects were familiarized with the investigators, experimental arrangements and exercise protocols by means of short preliminary practice runs.

#### 2.2.1 Anthropometric measurements

Whole body bioimpedance analysis was performed by a phase sensitive single frequency device (BIA 101 BIVA, Akern srl, Florence, Italy); fat mass (FM) and fat‐free mass (FFM) were determined by standard procedures (Lukaski, 1987).

#### 2.2.2 Resting energy expenditure

REE was measured by indirect calorimetry (Compher et al., 2006). The measurements were performed in the mid‐morning, 2–3 h after a light breakfast, during the first day of BR (BDC) and after 10 (BR_10_) and 21 (BR_21_, only in Y) days of BR. No significant exercise was performed by the subjects during the 18 h before the measurements, which were carried out in a dimly lit room, with standard temperature and humidity, isolated from disturbing noise. Pulmonary ventilation (${\dot{V}}_{E}$), tidal volume (*V*_T_), respiratory frequency (*f*_R_), end‐tidal O_2_ and CO_2_ partial pressures ($P_{\mathrm{ET}O_{2}}$ and $P_{\mathrm{ETC}O_{2}}$), pulmonary O_2_ uptake (${\dot{V}}_{O_{2}p}$) and CO_2_ output (${\dot{V}}_{CO_{2}}$) were determined breath‐by‐breath by a metabolic cart (Quark PFTergo, Cosmed, Rome, Italy), by utilizing a face mask and the ‘Resting Metabolic Rate’ software of the instrument. Expiratory flow measurements were performed by a turbine flowmeter, calibrated before each experiment by a 3‐L syringe at three different flow rates. Calibration of O_2_ and CO_2_ analysers was performed before each experiment by utilizing gas mixtures of known composition. Respiratory exchange ratio (RER) was calculated as ${\dot{V}}_{CO_{2}}$/${\dot{V}}_{O_{2}}$. During a 15‐min data acquisition period the subjects were in the supine position, and were instructed to remain as quiet as possible and to minimize movements. The software of the instrument averaged values every 30 s, and the data acquired during the last 10 min of the acquisition period were retained to calculate the REE (in kcal day^−1^) on the basis of ${\dot{V}}_{O_{2}}$, ${\dot{V}}_{CO_{2}}$ and RER measurements. Heart rate (HR) was simultaneously recorded by a chest band (S610i, Polar, Kempele, Finland).

#### 2.2.3 Resting muscle O_2_ uptake

Oxygenation changes in a superficial portion of the vastus medialis muscle were evaluated by time‐domain (TD) near‐infrared spectroscopy (NIRS) (Barstow, 2019; Delpy et al., 1988; Grassi & Quaresima, 2016; Lacerenza et al., 2025; Torricelli et al., 2014).

The NIRSBOX device (PIONIRS S.r.l., Milan, Italy) utilized in the present study is a commercial research‐grade TD‐NIRS tissue oximeter. The device is equipped with two picosecond lasers emitting near infra‐red light at 685 and 830 m, and a flexible, skin compatible, smart optical probe (G5 ‘Goccia’, PIONIRS S.r.l.) with 2.5 cm source–detector distance. The probe features a built‐in capacitive contact sensor to ensure correct application on the tissue and provide feedback to the researcher. Photons backscattered from the tissue are collected by a single photon detector, and their arrival times are recorded. Photon arrival times are used to reconstruct the photon time‐of‐flight distribution through time‐correlated single‐photon counting (TCSPC). Absorption (µ_a_) and reduced scattering (µ_s_′) coefficients are then retrieved by fitting the measured time of flight (TOF) distribution with a semi‐infinite homogeneous model based on the diffusion approximation (Contini et al., 1997). The differential pathlength factor (DPF) is also retrieved in real‐time from the features of the TOF distribution. Knowledge of these variables allows calculation of absolute values (expressed as µM) of [deoxy(Hb + Mb)], [oxy(Hb + Mb)] and ‘total’ [haem] present in the tissue ([oxy + deoxy (Hb + Mb)]) (Barstow, 2019; Torricelli et al., 2014). Skeletal muscle oxygenation, or ‘O_2_ saturation’) ($S_{mO_{2}}$) is then calculated as [oxy(Hb + Mb)]/[oxy + deoxy(Hb + Mb)], and expressed as a percentage.

The probe was firmly attached to the skin overlying the lower third of the vastus medialis muscle (∼10 cm above the knee joint), parallel to the major axis of the thigh, by a belt secured by Velcro straps and adhesive tape. The skin was carefully shaven before the experimentation. The site where the probe was placed was recorded using a skin marker and reproduced throughout the tests. Black cloth was put around the probe and the skin to prevent contamination from ambient light. The sampling frequency was set at 10 Hz. Skinfold thickness at the site of application of the probe was determined by a calliper (Gima, Milan, Italy). The average values of skin and subcutaneous tissue thickness were 6.0 ± 3.1 mm (range 2.8–10.9 mm) at BDC versus 6.5 ± 4.1 mm (range 1.9–13.9 mm) at POST BR in Y (*P* = 0.292), and 7.3 ± 1.6 mm (range 4.0–9.7 mm) at BDC *vs* 6.3 ± 2.6 mm (range 3.5–11.8 mm) at POST BR in E (*P* = 0.197).

Resting ${\dot{V}}_{O_{2}m}$ measurements at BDC and POST BR were performed with the subject sitting on the cycle ergometer, early in the afternoon (2–3 h after a light lunch), before any measurement involving a significant effort by the subject. Measurements were performed 2 days before the BR period (BDC) and the first day after the subjects rose from bed (POST BR). The subjects were instructed to place the leg on a wooden platform (height 10 cm), with the foot fixed to the pedal, and to keep the other leg relaxed (foot fixed to the pedal). At BR_10_ in Y (that is, *during* the BR period) measurements were performed with the subjects in supine position. ${\dot{V}}_{O_{2}m}$ was estimated by calculating the slope of the initial (∼1 min) linear decrease in $S_{mO_{2}}$ during an ∼5‐min ischaemic manoeuvre, performed for a different purpose (evaluation of the reoxygenation kinetics following ischaemia). Ischaemia was induced by rapid (less than 1 s) inflation of a pneumatic cuff, positioned at the inguinal crease of the thigh, to a suprasystolic pressure (∼300 mmHg) (DN 200/10/5 air compressor, Stanley, New Britain, CT, USA).

#### 2.2.4 Skeletal muscle biopsies and citrate synthase activity

Skeletal muscle biopsies were obtained from the vastus lateralis muscle under local anaesthesia (2% lidocaine). Biopsies were taken during the first day of BR (BDC) and during the last day of BR (BR_10_ in E and BR_21_ in Y). Following the application of anaesthetic, a 1.0–1.5 cm incision was made to the skin, subcutaneous tissue and muscle fascia, and the tissue sample was harvested with a Rongeur Conchotome (Zepf Instruments, Dürbheim, Germany). The collected muscle tissue was dissected free of fat and connective tissue and rapidly divided into several portions. One portion of the sample was immediately frozen in liquid nitrogen and stored at −80°C until determination of citrate synthase (CS) activity. Muscle samples were thawed and underwent a motor‐driven homogenization in a pre‐cooled 1 mL glass–glass potter (WHEATON^®^, DWK Life Sciences, Millville, NJ, USA). The muscle specimen was suspended 1:50 w/v in a homogenization buffer containing sucrose (250 mM), Tris (20 mM), KCl (40 mM) and EGTA (2 mM) with 1:50 v/v protease (P8340, Sigma‐Aldrich, St Louis, MO, USA) inhibitors. The specimen was homogenized in an ice‐bath with 20 strokes at 500 rpm; before the last hit, Triton X‐100 (0.1% v/v) was added to the solution. After this, the sample was left on ice for 30 min. The homogenate was centrifuged at 14,000 *g* for 10 min. The supernatant was used to evaluate protein concentration according to the method of Lowry et al. (1951). Protein extracts (5–10–15 µg) were added to each well of a 96‐well‐microplate along with 100 µL of 200 mM Tris, 20 µL of 1 mM 5,5′‐dithiobis‐2‐nitrobenzoate (DTNB), freshly prepared, 6 µL of 10 mM acetyl‐coenzyme A (acetyl‐CoA) and water to a final volume of 190 µL. A background ΔAbs, to detect any endogenous activity by acetylase enzymes, was recorded for 90 s with 10 s intervals at 412 nm at 25°C by a CLARIOstar Reader (BMG Labtech, Ortenberg, Germany). The ΔAbs was subtracted from the one given after the addition of 10 µL of 10 mM oxalacetic acid that started the reaction. All assays were performed at 25°C in triplicate on homogenates. Activity was expressed as nmol min^−1^ (mU) per mg of protein. The protocol was modified from Spinazzi et al. (2012) and Srere (1969).

### 2.3 Statistical analysis

Data are presented as means ± standard deviation (SD). A power analysis was performed a priori on the basis of the variance observed for the resting ${\dot{V}}_{O_{2}m}$ data in our previous study (Zuccarelli et al., 2021). After considering an α‐value of 0.05, and a 1 − β value of 0.80, an *n*‐value of 8 for each group was calculated to be enough to detect a statistically significant difference, if present (G*Power 3.1). Results were tested for normality using a Shapiro–Wilk test. The statistical significance of differences between two means was checked by a two‐tailed paired Student's *t‐*test or a Wilcoxon non‐parametric test for normally or non‐normally distributed data, respectively. The level of significance was set at *P *< 0.05. In order to check the statistical significance of differences among more than two means, a repeated measures analysis of variance (ANOVA) with the Greenhouse–Geisser correction was performed. When significant differences were found at ANOVA, a Tukey *post hoc* test was used to determine the location of the differences. Statistical analyses were carried out utilizing a commercially available software package (Prism 8.0; GraphPad Software, San Diego, CA, USA).

## 3 RESULTS

The main anthropometric and body composition data of the subjects are presented in Table 1. BM and body mass index decreased following BR in both groups, in association with a decreased fat free mass (FFM) and with an increased fat mass (FM).

**TABLE 1:** Some physical and anthropometric characteristics of the subjects.

	BDC	POST BR
Young		
Age (years)	23 ± 5	23 ± 5
Body mass (kg)	77.4 ± 4.4	74.3 ± 4.5***
Body height (m)	1.82 ± 0.07	1.83 ± 0.07*
BMI (kg m^−2^)	23.3 ± 2.1	22.3 ± 2.3***
FM (% BM)	14.4 ± 3.0	18.2 ± 4.0*
FFM (kg)	66.3 ± 5.4	60.8 ± 3.8***
Elderly		
Age (years)	69 ± 3	69 ± 3
Body mass (kg)	85.6 ± 12.3	83.9 ± 11.8***
Body height (m)	1.73 ± 0.06	1.73 ± 0.07
BMI (kg m^−2^)	28.7 ± 4.2	28.1 ± 4.0**
FM (% BM)	23.9 ± 7.1	26.1 ± 7.7 (*P* = 0.06)
FFM (kg)	64.6 ± 7.3	61.5 ± 6.1**

Mean ± SD values are shown. *P < 0.05, **P < 0.01, ***P < 0.001. BDC, baseline data collection; BM, body mass; BMI, body mass index; FFM, fat‐free mass; FM, fat mass; POST BR, after the end of the bed rest period.

**FIGURE 2:**
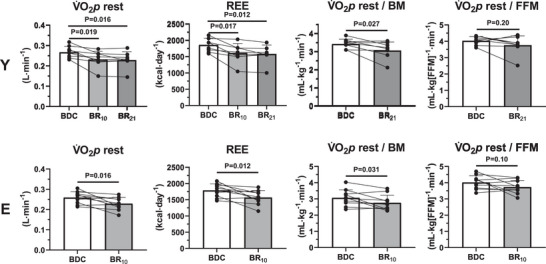
Mean (±SD) and individual values of resting pulmonary O_2_ uptake (${\dot{V}}_{O_{2}p}$) and resting energy expenditure (REE) at baseline data collection (BDC) and after 10 (BR_10_) and 21 (BR_21_) days of bed rest in the young (Y) and elderly (E) subjects. Resting ${\dot{V}}_{O_{2}p}$ data are expressed in L min^−1^, per unit of body mass (BM) (mL min^−1^ kg^−1^ BM) and per unit of fat free mass (mL min^−1^ kg^−1^ FFM). *P*‐values are also indicated. See text for further details.

Resting pulmonary ${\dot{V}}_{O_{2}}$ (${\dot{V}}_{O_{2}p}$) and the calculated daily REE (Figure 2) decreased in both groups following BR. In Y resting ${\dot{V}}_{O_{2}p}$ and REE were lower in BR_10_ and BR_21_ (about −14%) versus BDC; no differences were observed between values at BR_10_ and BR_21_. In E, the percentage decrease in resting ${\dot{V}}_{O_{2}p}$ and REE between BDC and BR_10_ was slightly less pronounced (−12%, −1.2% day^−1^) than that described for Y. The resting ${\dot{V}}_{O_{2}p}$ and REE decreases during the whole BR periods (21 days in Y, 10 days in E) were significant also after they were adjusted for the associated BM decrease, whereas the decreases did not reach statistical significance when values were normalized per FFM. In Y, normalizations of resting ${\dot{V}}_{O_{2}p}$ per unit of BM or FFM could not be carried out at BR_10_, since these measurements could not be taken at that time point (the subjects were in bed).

The data of the main cardiorespiratory variables determined during the experiments performed for the REE evaluation are presented in Table 2. The decreased REE at BR_21_ (Y) and BR_10_ (E) versus BDC was associated with decreases in resting ${\dot{V}}_{O_{2}p}$, ${\dot{V}}_{CO_{2}}$, RER (not significant in Y) and ${\dot{V}}_{E}$ (not significant in E), whereas no changes were observed for the other variables.

**TABLE 2:** Main cardiorespiratory variables determined during the measurements performed for the calculation of REE.

	BDC	BR_10_	BR_21_
Young			
REE (kcal day^−1^)	1853 ± 205	1606 ± 286*	1581 ± 267*
${\dot{V}}_{O_{2}}$ (L min^−1^)	0.268 ± 0.030	0.232 ± 0.040*	0.230 ± 0.040*
${\dot{V}}_{O_{2}}$ (mL kg min^−1^)	3.5 ± 0.3	—	3.1 ± 0.5*
${\dot{V}}_{CO_{2}}$ (L min^−1^)	0.223 ± 0.028	0.192 ± 0.043	0.185 ± 0.029*
RER	0.83 ± 0.06	0.83 ± 0.09	0.81 ± 0.07
${\dot{V}}_{E}$ (L min^−1^)	8.5 ± 0.8	7.3 ± 1.3*	7.6 ± 0.8***
*V*_T_ (L)	0.666 ± 0.116	0.630 ± 0.172	0.640 ± 0.211
*f*_R_ (breaths min^−1^)	13.4 ± 2.9	12.5 ± 3.7	12.8 ± 2.9
$P_{\mathrm{ET}O_{2}}$ (mmHg)	103.3 ± 3.1	102.8 ± 5.7	105.0 ± 4.9
$P_{\mathrm{ETC}O_{2}}$ (mmHg)	35.4 ± 3.4	36.7 ± 3.8	34.1 ± 3.1
HR (b min^−1^)	55 ± 10	53 ± 11	57 ± 14
$S_{pO_{2}}$ (%)	99 ± 1	98 ± 1	98 ± 1
Elderly			
REE (kcal day^−1^)	1788 ± 202	1568 ± 214*	
${\dot{V}}_{O_{2}}$ (L min^−1^)	0.269 ± 0.029	0.229 ± 0.031*	
${\dot{V}}_{O_{2}}$ (mL kg min^−1^)	3.1 ± 0.5	2.8 ± 0.5*	
${\dot{V}}_{CO_{2}}$ (L min^−1^)	0.214 ± 0.028	0.181 ± 0.027*	
RER	0.83 ± 0.06	0.79 ± 0.05**	
${\dot{V}}_{E}$ (L min^−1^)	9.6 ± 1.7	9.0 ± 1.5	
*V*_T_ (L)	0.825 ± 0.499	0.667 ± 0.248	
*f*_R_ (breaths min^−1^)	14.4 ± 5.6	15.1 ± 5.4	
$P_{\mathrm{ET}O_{2}}$ (mmHg)	108.8 ± 3.7	111.1 ± 4.7*	
$P_{\mathrm{ETC}O_{2}}$ (mmHg)	30.6 ± 3.4	28.2 ± 4.2	
HR (b min^−1^)	59 ± 7	59 ± 11	
$S_{pO_{2}}$ (%)	97 ± 2	97 ± 2	

Mean ± SD values are shown. * *P* < 0.05, ** *P* < 0.01, *** *P *< 0.001. BDC, baseline data collection; BR_10_, 10th day of bed rest; BR_21_, 21st day of bed rest; *f*_R_, breathing frequency; HR, heart rate; $P_{\mathrm{ETC}O_{2}}$, end‐tidal CO_2_ partial pressure; $P_{\mathrm{ET}O_{2}}$, end‐tidal O_2_ partial pressure; REE, resting energy expenditure; RER, respiratory exchange ratio; $S_{pO_{2}}$, arterialized blood O_2_ saturation by pulse oximetry; ${\dot{V}}_{CO_{2}}$, CO_2_ output; ${\dot{V}}_{E}$, pulmonary ventilation; ${\dot{V}}_{O_{2}}$, pulmonary O_2_ uptake; *V*_T_, tidal volume.

$S_{mO_{2}}$ values obtained at rest in the various experimental conditions were in Y 73.6 ± 3.5% in BDC, 77.5 ± 2.5% in BR_10_ and 73.4 ± 3.4% in BR_21_. Resting $S_{mO_{2}}$ values in E were 73.5 ± 1.9% in BDC and 72.4 ± 3.2% in BR_10_.

In Table 3 the values of total (oxygenated + deoxygenated) haemoglobin + myoglobin concentrations ([oxy + deoxy(Hb + Mb)]), obtained at rest and during ischaemia, are presented. At BDC (*P* ≤ 0.001) and at BR_10_ (*P* ≤ 0.007) values were significantly higher in Y versus E. During BR, values in Y significantly decreased (*P* ≤ 0.038), whereas they did not change in E (*P* ≥ 0.277). In both groups of subjects and in all experimental conditions [oxy + deoxy(Hb + Mb)] values at rest were not significantly different from those obtained during the ischaemic manoeuvre.

**TABLE 3:** Total (oxygenated + deoxygenated) haemoglobin + myoglobin concentration ([oxy + deoxy(Hb + Mb)]) values, expressed as µM, obtained by time‐domain near‐infrared spectroscopy (TD‐NIRS) at rest and during ischaemia in the young (Y) subjects, at baseline data collection (BDC), after 10 (BR_10_) and after 21 (BR_21_) days of bed rest, and in the elderly subjects (E) at BDC and at BR_10_.

	Young			Elderly	
	BDC	BR_10_	BR_21_	BDC	BR_10_
Rest	148.5 ± 28.4	123.2 ± 21.7*	122.1 ± 21.8*	88.5 ± 29.4^###^	85.0 ± 27.9^##^
Ischaemia	151.7 ± 31.4	124.1 ± 23.1*	123.4 ± 22.0*	91.2 ± 29.1^###^	86.5 ± 26.8^##^

Values are presented as means ± SD. See text for further details. *P < 0.05 vs. BDC in Y; ^##^ *P *< 0.01, ^###^ *P *< 0.001 vs. Y at corresponding time point.

**FIGURE 3:**
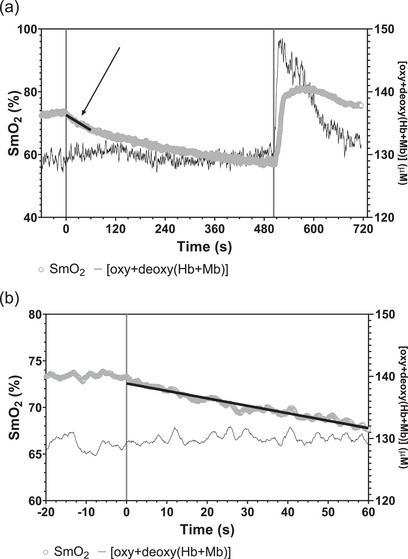
(a)Time courses during a transient ischaemia (vertical lines indicate start and end of the ischaemic period) of vastus medialis muscle O_2_ saturation ($S_{mO_{2}}$) and total [haem] (oxygenated + deoxygenated haemoglobin + myoglobin concentrations ([oxy + deoxy(Hb + Mb)]) values, determined by time‐domain near‐infared spectroscopy. The regression line utilized to determine the initial slope of $S_{mO_{2}}$ decrease and to calculate resting muscle O_2_ uptake is also shown, and is indicated by the arrow. See text for futher details. (b) The same data presented in panel (a) are shown. The time axis is expanded, with respect to panel (a), in order to better describe the time courses of the variables during the first minute of ischaemia, in which the linear slope of $S_{mO_{2}}$ versus time was calculated in order to estimate resting ${\dot{V}}_{O_{2}m}$. See text for further details.

A typical example of the $S_{mO_{2}}$ and [oxy + deoxy(Hb + Mb)] time courses during a transient ischaemia is shown in Figure 3a, b. In Figure 3b the time axis is expanded in order to better describe the time course of the variable during the first minute of ischaemia, in which the linear slope of $S_{mO_{2}}$ versus time was calculated in order to estimate resting ${\dot{V}}_{O_{2}m}$. The regression line utilized to calculate the slope of is also shown.

**FIGURE 4:**
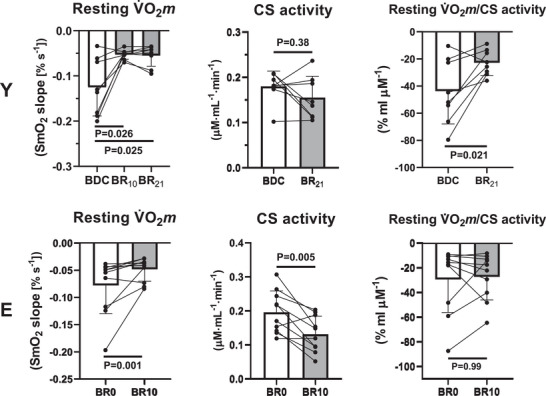
Mean (±SD) and individual values of resting muscle O_2_ uptake (${\dot{V}}_{O_{2}m}$), citrate synthase (CS) activity and resting ${\dot{V}}_{O_{2}m}$ /CS at baseline data collection (BDC) and the first day after the end of the BR period (POST BR), in the young (Y) and elderly (E) subjects. *P*‐values are also indicated. See text for further details.

Data on resting ${\dot{V}}_{O_{2}m}$ and citrate synthase (CS) activity are presented in Figure 4. Resting ${\dot{V}}_{O_{2}m}$ decreased during BR both in Y (−45%, −2.1% day^−1^; *P* = 0.025) and in E (−29%, −2.9% day^−1^; *P* = 0.001), following 21 and 10 days of exposure. In Y the whole resting ${\dot{V}}_{O_{2}m}$ decrease occurred during the first 10 days of BR, that is in BR_10_ versus BDC (−44%, −4.4% day^−1^). CS activity decreased at BR_10_ versus BDC in E (−31%; *P* = 0.005), whereas in Y the CS activity decrease in BR_21_ versus BDC (−12%) was not statistically significant (*P* = 0.38). After resting ${\dot{V}}_{O_{2}m}$ data were normalized for CS activity, they were significantly lower in BR_21_ versus BDC in Y (*P* = 0.021), whereas no difference was observed between BR_10_ and BDC in E (*P* = 0.99).

**FIGURE 5:**
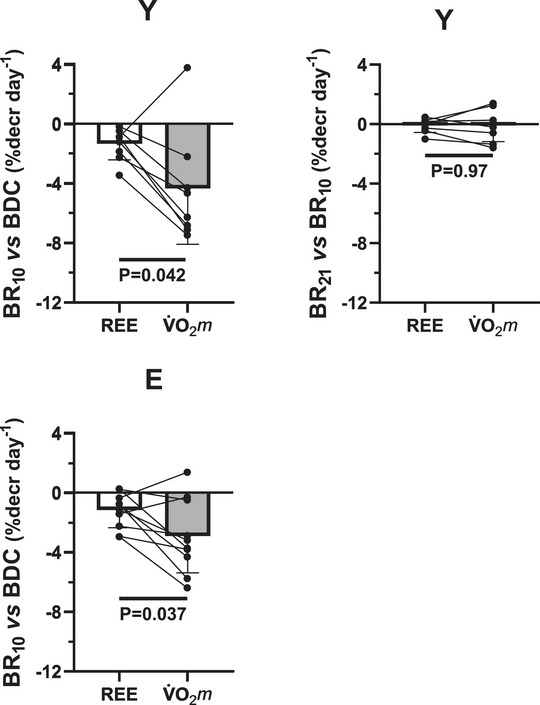
Percentage decreases, expressed per day of BR exposure, are presented for resting energy expenditure (REE) and resting ${\dot{V}}_{O_{2}m}$, for Y and E. For Y, data are presented following the first 10 days of BR (BR_10_ vs. BDC) and following the last 11 days of BR (BR_21_ vs. BR_10_).

In Figure 5 the percentage decreases per day of BR exposure are presented for REE and resting ${\dot{V}}_{O_{2}m}$, for Y and E. For Y, data are presented following the first 10 days of BR (BR_10_ vs. BDC) and following the last 11 days of BR (BR_21_ vs. BR_10_). For both variables percentage decreases per day were more pronounced in Y versus E. Both in Y and in E percentage decreases per day were more pronounced for resting ${\dot{V}}_{O_{2}m}$ versus REE. In Y, for both variables the decreases occurred only during the first 10 days of BR.

## 4 DISCUSSION

The main hypothesis of the present study was confirmed: during BR the decrease in resting ${\dot{V}}_{O_{2}m}$, already described in a previous study by our group (Zuccarelli et al., 2021) in a different cohort of young subjects following a 10‐day BR, was accompanied by a decreased whole‐body REE. This occurred both in Y and in E subjects. REE values at BDC were in agreement with those usually presented in the literature (see e.g. Heymsfield et al., 2021). The decrease in REE during the first 10 days of BR was, percentage‐wise, slightly greater in Y (about −14%) versus E (about −12%); from BR_10_ to BR_21_ the REE decrease in Y was very small. To the best of our knowledge only two previous studies (Downs et al., 2020; Noone et al., 2023) reported data of REE during (head‐down) BR studies, of longer duration (60–70 days). In the control conditions (no nutritional interventions) of both those studies (Downs et al., 2020; Noone et al., 2023) the REE decreases were less pronounced compared to that observed in the present study. No clear explanation can be put forward to explain this discrepancy. The fact that in our study the BR was horizontal, whereas Downs et al. (2020) and Noone et al. (2023) utilized the head‐down tilt approach, should not have influenced REE. It may be hypothesized that during prolonged BR (60–70 days; Downs et al., 2020; Noone et al., 2023) the decrease in REE is attenuated compared to that observed during the first few weeks of exposure. It should also be considered that energy expenditure is closely regulated by both energy intake and overall energy balance (Fernandez‐Verdejo et al., 2024). Caloric restriction, for example, reduces REE (see e.g. Poole & Henson, 1988). Different energy intake protocols used across studies could influence REE measurements, as energy deficits or surpluses can alter metabolic rates. In our study the specific dietary conditions and energy intake (the diet was eucaloric and it was individually adjusted for the reduced metabolic rate) should not have affected REE.

As mentioned in the Introduction, a decreased REE could be beneficial during long‐duration spaceflights, by lowering rates of crewmember consumable use (food, water and O_2_) and CO_2_ production (Regan et al., 2020). This concept led Regan et al. (2020) to envisage the induction of a state of shallow metabolic depression during spaceflights, which could reduce REE by ∼20%. It is interesting to observe that the decreased REE (−15%) observed in Y following 21 days of BR lies reasonably close to this value. Thus, inactivity during spaceflights could be enough to obtain the desired decrease in REE. Inactivity during spaceflights (in other words, no exercise countermeasures), however, would have profound and negative consequences for several organs, systems and functions of the body, exposing the crewmember's health to significant and unacceptable risks. Spaceflights are indeed nowadays characterized by intensive programmes of in‐flight exercise, comprising moderate and/or high‐intensity aerobic exercise, associated with resistance exercise (Ade et al., 2017; Scott et al., 2023), aimed at attenuating the negative effects of inactivity and microgravity on organs, systems and functions. A balance between inactivity (aimed at obtaining a decrease in REE) and exercise (aimed at protecting the function of organs and the health of the astronauts) seems difficult to obtain, and the issue deserves further investigation.

A reduced REE during BR or inactivity/muscle disuse could have also other negative consequences on the general health status of subjects, and could also impair mission readiness on arrival on planetary habitats. As mentioned in the Introduction, an elevated REE is generally considered beneficial in terms of the prevention of obesity and in the maintenance of body mass (Heymsfield et al., 2021). Thus, a lower REE could have negative consequences for the health status of subjects, by altering body mass homeostasis and increasing the risk of decreased insulin sensitivity and metabolic diseases.

In any case, apart from the issue of the practical positive effects of a decreased REE during spaceflights (Regan et al., 2020), and of the potentially negative consequences for the health status, the results of the present study are relevant in terms of the mechanisms regulating resting metabolic rate, particularly in skeletal muscle, during periods of inactivity/muscle disuse.

The REE decrease observed, by indirect calorimetry, was associated with a resting ${\dot{V}}_{O_{2}m}$ decrease, obtained by a completely different experimental approach. In the present study, resting ${\dot{V}}_{O_{2}m}$ was estimated on the basis of the linear decrease of the TD‐NIRS‐obtained muscle ‘O_2_ saturation’ signal ($S_{mO_{2}}$) during a period of transient limb ischaemia, taken as an indirect index of ${\dot{V}}_{O_{2}m}$ (see other studies performed by applying the same approach, although with different instruments, such as Adami & Rossiter, 2018; Barstow, 2019; Hamaoka et al., 1996; Ryan et al., 2012; Van Beekvelt et al., 2001; Zuccarelli et al., 2021, 2020). TD‐NIRS uses short laser pulses and time‐resolved detection to measure photon travel time, allowing the separation of light absorption and scattering effects, as well as the measurement of the pathlength covered by photons. By doing so, TD‐NIRS allows calculation of absolute concentrations of oxygenated and deoxygenated cromophores (Hb and Mb), and expression $S_{mO_{2}}$ as a percentage. It should be noted, however, that it is not possible to express ${\dot{V}}_{O_{2}m}$ in absolute values, as a consequence of the uncertainties related to the relative contribution of Hb (four O_2_ carried by the chromophore in the oxygenated state) and Mb (only one O_2_ carried by the chromophore in the oxygenated state) to the NIRS signal (Barstow, 2019; Porcelli et al., 2023).

In the present study the resting ${\dot{V}}_{O_{2}m}$ decrease was greater, percentage‐wise, than that determined for whole‐body REE. This occurred both in Y and in E (see Figure 5). In other words, the decrease in resting ATP supply from oxidative metabolism, during BR was more pronounced in skeletal muscles versus that observed in other tissues and organs. This is not surprising, considering the marked ‘plasticity’ of skeletal muscle with respect to external perturbations such as training, detraining, microgravity and disuse (see e.g. Delfinis et al., 2025). In Y the REE and resting ${\dot{V}}_{O_{2}m}$ decreases occurred early, during the first 10 days of BR, with no further decreases observed between days 10 and 21.

Inactivity/muscle disuse can cause a decrease in muscle mass and function (Booth et al., 2017; Monti et al., 2021; Motanova et al., 2024; Sarto et al., 2023), in mitochondrial content and function (Hood et al., 2019), an increase in mitochondrial fission and decrease in fusion (Picard & Turnbull, 2013), as well as a decreased maximal ADP‐stimulated mitochondrial respiration (‘OXPHOS capacity’), at least for BR periods lasting several weeks (Eggelbush et al., 2024; Noone et al., 2023). On the other hand, although the data are somehow controversial (see e.g. Dirks et al., 2020), during shorter BR periods (up to at least 10 days) OXPHOS capacity would be preserved (Eggelbush et al., 2024; Zuccarelli et al., 2021).

CS is a key enzyme in the Krebs cycle and is widely used as a marker for mitochondrial activity and mitochondrial volume in muscle tissue (Larsen et al., 2012). Under BR conditions a drop in CS activity has been observed in the early phases of BR, within 4 (Larsen et al., 2018) or 7 days (Dirks et al., 2016; Ringholm et al., 2011). In the present study we observed a significant decrease in CS activity in E, whereas in Y the decrease did not reach statistical significance. A significant dispersion of data was indeed observed in Y at BR_21_, with four participants showing a decrease, whereas the remaining four participants showed no change or even an increase. No methodological problems could be identified which could explain the significant data variability. In any case, it can be hypothesized that the decreased CS activity may result from increased protein degradation, which has been shown to occur with BR (Brocca et al., 2012; Standley et al., 2020), or from a decreased demand for ATP, associated with inactivity, and leading to ATP accumulation, which could allosterically determine a decrease in CS content (Edwards et al., 2021), and/or to post‐translational modifications.

The decrease in resting ${\dot{V}}_{O_{2}m}$ observed in the present study could represent a response aimed at decreasing, as much as possible, ATP accumulation, or an excessive ROS production, in a response similar to that observed in hibernating animals (De Napoli et al., 2025; Staples et al., 2022). As proposed by Delfinis et al. (2025), the decreased resting ${\dot{V}}_{O_{2}m}$ and REE observed in the present study would represent a ‘recalibration’ of ATP supply to the reduced ATP demand associated with bed rest, possibly aimed at preventing excessive ROS production and at mitigating muscle atrophy. In the present study the resting ${\dot{V}}_{O_{2}m}$ decrease in E can be fully explained by the associated CS activity decrease, taken as an estimate of mitochondrial mass (Larsen et al., 2012). In Y, on the other hand, as mentioned above, CS activity decrease did not reach statistical significance. Thus, ‘qualitative’ (not merely ‘quantitative’, i.e. related to mitochondrial mass) factors could be responsible in Y for the observed resting ${\dot{V}}_{O_{2}m}$ decrease. Incidentally, a reduced resting ${\dot{V}}_{O_{2}m}$ in the presence of an unchanged CS activity was already described by our group in young subjects following a shorter BR period (Zuccarelli et al., 2021).

In terms of ‘qualitative’ factors, it may be hypothesized that the decreased ATP turnover during BR may derive, at least in part, from a greater percentage of myosin molecules being ‘parked’, as a consequence of the chronic inactivity/disuse, in the super‐relaxed functional state (SRS), in which folded‐back myosin heads lie plastered in the shaft of the thick filament, and have an ultra‐low ATP turnover (De Napoli et al., 2025; Lewis & Ochala, 2023; Nag & Trivedi, 2021; Nogara et al., 2016; Stewart et al., 2010). The SRS state may represent an energy‐saving mechanism, aimed at obtaining a significant reduction in the resting metabolism of an organism, by avoiding unnecessary ATP‐consuming cycles. The mechanism may be vital for survival in hibernating animals (De Napoli et al., 2025; Lewis et al., 2024), possibly aimed at mitigating muscle wasting (De Napoli et al., 2025). The role played by the SRS mechanisms, as well as by others (e.g. uncoupling proteins, altered autonomic control, thyroid hormones) during inactivity or bed rest in humans obviously needs to be demonstrated experimentally. Other factors, potentially responsible for the reduced mitochondrial mass and function and the lower resting ${\dot{V}}_{O_{2}m}$ during BR, could be related to ‘upstream’ impairments in the O_2_ transport mechanism occurring during BR, demonstrated in previous studies by our group and by others, such as impaired cardiac (see e.g. Baldassarre et al., 2022) and microvascular/endothelial (Zuccarelli et al., 2021) functions. Changes of REE in the opposite direction (e.g. increases) have been described during altitude exposure (Kuikman et al., 2025). In these cases, the effects on REE could be related to mechanisms such as the hypoxia‐inducible factor and an increased sympathetic stimulation.

The TD‐NIRS oximeter utilized in the present study allows calculation (differently from the more frequently utilized continuous‐wave (CW)‐NIRS instruments; Barstow, 2019; Grassi & Quaresima, 2016) of absolute values of ‘[total haem]’ ([oxy + deoxy(Hb + Mb)]) (Barstow, 2019) in the tissue of interest (see data in Table 3). Interestingly, [oxy + deoxy(Hb + Mb)] was higher in Y than in E, and it decreased in Y during BR. A [oxy + deoxy(Hb + Mb)] decrease could be attributed to decreased capillarity, decreased microvascular haematocrit, fibre‐type difference with a decreased presence of oxidative fibres (rich in Mb), and/or decreased [Mb] in the fibres.

In the present study the very stringent experimental schedules inherently associated with BR studies, in which the experiments performed by various groups of researchers must be tightly packed in relatively limited time windows, did not allow us to follow the very strict criteria utilized to define a ‘basal metabolic rate’ (Compher et al., 2006; Heymsfield et al., 2021), with specific reference to previous exercise and previous food consumption. Thus, we decided to utilize the less stringent definition of ‘resting energy expenditure’ (Heymsfield et al., 2021). The experimental conditions were, however, rigorous (see ‘Methods’) and highly reproducible before and after BR.

Another limitation is presented by the fact that only male subjects were tested. Recruiting also female participants would have inevitably reduced the statistical power of comparisons between sex groups, thus requiring a greater total number of participants, which is not usually possible in BR studies, mainly owing to logistic and financial constraints. These constraints led us to recruit only male subjects. We recognize this limits the generalizability of the observed results, and future BR studies will have to be conducted, if feasible, also (or only) on females.

The relatively limited number of subjects impacted our study also from another perspective. In some instance the observed differences of variables did not reach statistical significance. At least in part this could be attributed to the relatively low number of subjects. The present study was adequately powered to detect statistically significant differences of the main variable of interest, that is, resting ${\dot{V}}_{O_{2}m}$. It should also be noted that both in the present study and in other recent papers by our group deriving from another recent BR campaign (see e.g. Baldassarre et al., 2022; Monti et al., 2021; Zuccarelli et al., 2021, 2025), an *n*‐value of 8–10 allowed detection of statistically significant differences of variables of specific interest, amongst which was resting ${\dot{V}}_{O_{2}m}$ (Zuccarelli et al., 2021).

The relatively low number of subjects precludes also the possibility of specifically investigating the interindividual heterogeneity of responses to microgravity for the various variables (see e.g. Salvadego et al., 2021), which could be of interest in bed rest studies, particularly in relation to the selection of crewmembers.

The duration of the BR was different in the Y (21 days) and E (10 days) groups. BR studies are particularly difficult to perform in elderly subjects, mainly for ethical reasons associated with the multiple risks of medical complications deriving from the prolonged inactivity. This makes BR studies in the elderly very rare. Ten days was the longest period considered to be ethically feasible in E. In order to compare the rate of changes of variables during different durations of BR, we calculated, for same variables, the mean percentage change per day. We recognize, however, that the different durations of BR in the two groups is a limitation, which did not allow us to make inferences on the linearity or non‐linearity of the observed changes as a function of time.

Resting ${\dot{V}}_{O_{2}m}$ measurements in the present study were performed by TD‐NIRS on the vastus medialis muscle, whereas in our previous work (Zuccarelli et al., 2021) measurements were performed by CW space‐resolved NIRS (Grassi & Quaresima, 2016, Barstow, 2019) on the vastus lateralis. The muscle biopsies for CS determination were performed, in both studies, on the vastus lateralis. The consequences for data interpretation should, however, be minimal, considering that the fibre type distribution in the two muscles is very similar (Saltin & Gollnick, 1983).

We recognize that the present study is relatively weak in terms of the investigation of molecular mechanisms potentially responsible for the observed resting ${\dot{V}}_{O_{2}m}$ decrease. These mechanisms will have to be investigated in future BR campaigns, which are being organized. Also the potential consequences for insulin resistance (see e.g. Dirks et al., 2020, 2016; Pišot et al., 2016) were not investigated.

To conclude, during BR (21 days in young subjects, 10 days in elderly subjects) a decrease in resting ${\dot{V}}_{O_{2}m}$, already described by our group in young subjects following a previous campaign of 10‐day BR (Zuccarelli et al., 2021), was accompanied by a decreased whole‐body REE. This occurred both in young and in elderly subjects. Even though far less pronounced compared to the typical reduction of REE observed in obligate hibernating animals (De Napoli et al., 2025; Staples et al., 2022), the observed inactivity‐related decrease in ${\dot{V}}_{O_{2}m}$ and REE could represent a ‘recalibration’ of ATP supply to the reduced ATP demand, possibly aimed at preventing excessive ROS production and at mitigating muscle atrophy (Delfinis et al., 2025). The decreased REE would also go in the direction of mitigating numerous biological and logistic challenges of prolonged spaceflights (Regan et al., 2020). On the other hand, the inactivity‐related decrease in REE would have negative consequences for the health status of subjects, by altering body mass homeostasis and increasing the risk of metabolic diseases. These negative consequences would be of particular interest for subjects exposed, on Earth, to periods of hospitalization, recurrent illnesses, environmental constraints leading to inactivity and muscle disuse.

## AUTHOR CONTRIBUTIONS

Bruno Grassi conceived the study; Bruno Grassi, Marco Narici and Rado Pišot obtained the funding; Giovanni Baldassarre, Simone Porcelli, Lucrezia Zuccarelli, Boštjan Šimunič, Rado Pišot and Marco Narici coordinated the conduct of the experiments; Giovanni Baldassarre, Lucrezia Zuccarelli, Simone Porcelli, Michele Lacerenza, Alessio Marciano, Chiara Motto, Lorenza Brocca, Noemi Arboit, Laura Gobbo, Giulia Sanguin, Mladen Gasparini participated in the experiments, data analysis and interpretation; Bruno Grassi, Giovanni Baldassarre, Simone Porcelli and Lucrezia Zuccarelli wrote the first draft of the manuscript. All authors have read and approved the final version of this manuscript and agree to be accountable for all aspects of the work in ensuring that questions related to the accuracy or integrity of any part of the work are appropriately investigated and resolved. All persons designated as authors qualify for authorship, and all those who qualify for authorship are listed.

## CONFLICT OF INTEREST

M.L. is founder of PIONIRS s.r.l. The other authors declare no competing interests.

## Data Availability

The datasets used and analysed during the current study are available from the corresponding author on reasonable request.
